# Survival in non-small cell lung cancer patients with versus without prior cancer

**DOI:** 10.1038/s41598-023-30850-2

**Published:** 2023-03-15

**Authors:** Akira Sato, Toshitaka Morishima, Masato Takeuchi, Kayo Nakata, Koji Kawakami, Isao Miyashiro

**Affiliations:** 1 Cancer Control Center, Osaka International Cancer Institute, 3-1-69 Otemae, Chuo-ku, Osaka, 541-8567 Japan; 2 Department of Pharmacoepidemiology, Graduate School of Medicine and Public Health, Kyoto University, Yoshidakonoe-cho, Sakyo-ku, Kyoto, Japan

**Keywords:** Cancer, Diseases, Medical research, Oncology, Risk factors

## Abstract

Clinical trials on cancer treatments frequently exclude patients with prior cancer, but more evidence is needed to understand their possible effects on outcomes. This study analyzed the prognostic impact of prior cancer in newly diagnosed non-small cell lung cancer (NSCLC) patients while accounting for various patient and cancer characteristics. Using population-based cancer registry data linked with administrative claims data, this retrospective cohort study examined patients aged 15–84 years diagnosed with NSCLC between 2010 and 2015 in Japan. Cox proportional hazards models were used to estimate the hazard ratios (HRs) and 95% confidence intervals (CIs) of all-cause mortality in patients with versus without prior cancer. The analysis was stratified according to NSCLC stage and diagnostic time intervals between prior cancers and the index NSCLC. We analyzed 9103 patients (prior cancer: 1416 [15.6%]; no prior cancer: 7687 [84.4%]). Overall, prior cancer had a non-significant mortality HR of 1.07 (95% CI: 0.97–1.17). Furthermore, prior cancer had a significantly higher mortality hazard for diagnostic time intervals of 3 years (HR: 1.23, 95% CI: 1.06–1.43) and 5 years (1.18, 1.04–1.33), but not for longer intervals. However, prior cancer in patients with more advanced NSCLC did not show a higher mortality risk for any diagnostic time interval. Smoking-related prior cancers and prior cancers with poorer prognosis were associated with poorer survival. NSCLC patients with prior cancer do not have an invariably higher risk of mortality, and should be considered for inclusion in clinical trials depending on their cancer stage.

## Introduction

The improved survival of cancer patients has inadvertently led to an increase in the number of persons with multiple primary cancers^1–4^. Despite the growing prevalence of newly diagnosed cancer patients with a history of cancer (e.g., 18.7% in the US and 11.1% in Japan)^4,5^, such patients are often excluded from clinical trials due to concerns that their presence may unduly affect outcomes and distort conclusions^6–11^. Overall, 80% of trials excluded lung cancer patients with prior cancer. The estimated proportion of patients with lung cancer excluded because of prior cancer ranged from 0 to 18%^7^. The omission of these patients may limits the generalizability of findings from clinical trials and observational research, resulting in a dearth of evidence on new treatment modalities for cancer survivors^6–11^.

Previous studies have produced conflicting findings on the prognostic impact of prior cancer in newly diagnosed lung cancer patients. While some studies have reported that prior cancer did not reduce survival in early-stage, locally advanced, or advanced lung cancer patients^12–14^; others have noted that the prognostic impact of prior cancer can vary according to lung cancer stage^15–18^. In contrast, several studies have indicated that prior cancer negatively impacts survival in lung cancer patients after adjusting for clinical prognostic factors^19–21^. Accordingly, there is a need for multicenter studies that explore the impact of prior cancer on survival in large cancer populations while adjusting for known clinical prognostic factors. Furthermore, studies should also consider lung cancer stage, the diagnostic time intervals between prior cancers and new cancers, and the diverse characteristics of prior cancers, including stage, smoking-relatedness^22,23^.

More research is required to facilitate the evidence-driven development of eligibility criteria for clinical cancer research. To contribute to this evidence base, this study was conducted to provide clinically comparable estimates of the prognostic impact of prior cancer in newly diagnosed non-small cell lung cancer (NSCLC) patients in a Japanese prefecture with consideration to patient characteristics, cancer characteristics, and diagnostic time interval.

## Materials and methods

### Study design and data sources

This multicenter retrospective cohort study was conducted on newly diagnosed NSCLC patients to compare their mortality risk with and without prior cancer using a database that linked population-based cancer registry data with administrative claims data.

Cancer registry data were acquired from the Osaka Cancer Registry (OCR), which was founded in 1962 with the aim of registering and monitoring all malignant tumors and benign intracranial tumors in residents of Osaka Prefecture—Japan’s third largest metropolis^24^. The registry data include each patient’s age at diagnosis, sex, method of cancer detection, cancer site, histology, stage, treatment, and vital status information (verified through death certificates and official resident registries). The OCR contains high-quality data that have been used in the International Agency for Research on Cancer’s “Cancer Incidence in Five Continents” Volumes III to XI^25^.

The claims data were generated by acute care hospitals under Japan’s Diagnosis Procedure Combination Per-Diem Payment System for the purpose of reimbursement from insurers. These data incorporate clinical summaries and detailed claims records of treatments that are not included in cancer registry data^26^.

Linked data were collected from 35 cancer care hospitals with cooperation from the Council for Coordination of Designated Cancer Care Hospitals (Osaka, Japan). These 35 hospitals treat approximately half of all cancer patients within Osaka Prefecture. The cancer registry data and claims data were linked at the patient level, and the record linkage rate was estimated to be 98%^18,27,28^.

### Study population

The study population comprised patients who received a new diagnosis of NSCLC between 2010 and 2015, which was designated the index cancer for this study. Patients who fulfilled the following criteria were included in the analysis: (1) aged 15–84 years at the NSCLC diagnosis, (2) pathological diagnosis of NSCLC, (3) survived for 3 months or more after the NSCLC diagnosis, (4) claims data could be linked with OCR data, and (5) registration in the OCR through sources other than death certificate only. Lung cancer was identified using the ICD-10 code of C34.x, and NSCLC histology was determined using the relevant International Classification of Diseases for Oncology, Third Edition morphological codes. The study patients were identified as those with a diagnosis of adenocarcinoma (morphological codes: 8140, 8211, 8230–8231, 8250–8260, 8323, 8480–8490, 8550–8552, 8570–8574, 8576), squamous cell carcinoma (8041–8045), adenosquamous carcinoma (8560), large cell carcinoma (8010–8012, 8014–8031, 8035, 8310), or NSCLC-not otherwise specified (8046). We excluded patients aged 85 years or older with reference to the previous clinical trials^29,30^. Patients who were diagnosed with the index NSCLC were followed for a minimum of 3 years after lung cancer diagnosis.

We divided the patients into 2 groups: a Prior Cancer Group (patients with a history of prior cancer) and a No Prior Cancer Group (those without any history of prior cancer). Prior cancer was defined as the most recent cancer diagnosed before the index NSCLC, and was identified based on multiple cancer records in the OCR in accordance with the guidelines of the International Agency for Research on Cancer and the International Association of Cancer Registries^31^. We obtained information on all prior cancers that were diagnosed between 1975 and 2015 from the OCR database.

Multiple cancers of the same site in a patient were integrated into a single cancer based on stage (most advanced) and order of occurrence (most recent). We identified 44 prior cancer sites using their corresponding ICD-10 codes (Supplementary Table 1)^32^.

### Study outcome

The study outcome measure was overall survival, which was calculated from the date of the index NSCLC diagnosis to the date of all-cause death or the date of censoring (i.e., last confirmed survival date).

### NSCLC characteristics

For each patient, we analyzed age, sex, method of cancer detection, histology, stage, treatment, use of tyrosine kinase inhibitors targeting epidermal growth factor receptor or anaplastic lymphoma kinase in any line of treatment, current or past smoking status, and diagnosis year for the index NSCLC. The methods of cancer detection included screening and medical check-up, incidental detection during follow-up examination for another disease, and other/unknown (mostly involving detection based on subjective symptoms)^33^. NSCLC stage at diagnosis was specified according to the Surveillance, Epidemiology, and End Results (SEER) system, and included localized, regional, distant, and other/unknown^34^. Treatment included radiotherapy only, chemotherapy only, chemoradiotherapy, surgery only, surgery plus chemotherapy and/or radiotherapy, and other/unknown (including no treatment).

We used the Barthel Index to measure performance in activities of daily living at the index NSCLC diagnosis as a substitute for the Eastern Cooperative Oncology Group-Performance Status (ECOG-PS)^28^. Patients were defined as having severe dependence for Barthel Index scores of 35 or lower (corresponding to ECOG-PS 3 or 4), moderate dependence for scores of 40–55 (ECOG-PS 2), and mild or no dependence for scores of 60 or higher (ECOG-PS 0 or 1)^35^.

Body mass index at the index NSCLC diagnosis was categorized into underweight (< 18.5 kg/m^2^), normal weight (18.5–24.9 kg/m^2^), overweight (25–29.9 kg/m^2^), and obese (≥ 30 kg/m^2^)^28^.

The Quan version of the Charlson Comorbidity Index (CCI) scores^18,36^ were categorized into 0, 1, and 2 or more^12^. Primary and metastatic solid tumors were excluded from the CCI scores due to their potential overlap with the prior and index cancers. Furthermore, interstitial lung disease (ICD-10 code: J84.x) was analyzed as an independent covariate due to its strong prognostic implications in lung cancer^37^.

Finally, we analyzed the Area Deprivation Index of each patient’s area of residence as a socioeconomic indicator^38^. Area Deprivation Index scores were divided into quartiles, and categorized from Q1 (least deprived) to Q4 (most deprived). Missing values were categorized as “unknown”.

### Prior cancer characteristics

For patients in the Prior Cancer Group, we calculated the number, the stage, and the sites of prior cancers, as well as the diagnostic time interval between the most recent prior cancer and the index NSCLC. Prior cancers were categorized according to the following 3 characteristics: smoking-relatedness, prognosis, and SEER summary stage at diagnosis. Smoking-related prior cancers included cancers of the mouth, pharynx, larynx, lung, esophagus, stomach, liver, pancreas, kidney, urinary bladder (renal pelvis, ureter, or bladder), colorectum, uterine cervix, and acute myeloid leukemia^19,39–42^. All other cancers were regarded as non–smoking-related cancers. Next, prognoses of the prior cancers were categorized based on their survival rates; cancers with better prognosis and cancers with poorer prognosis were defined as those with ≥ 50% and < 50%, respectively, of the median 10-year relative survival rate (Supplementary Table 2)^43^. All other cancers with unknown survival rates were categorized as “unknown”.

### Statistical analysis

Categorical variables were analyzed as proportions, and continuous variables were analyzed as median (interquartile range).

For the survival analysis, we excluded synchronous prior cancers from the Prior Cancer Group due to their possible influence on the timing and method of treatment. Using the criteria proposed by Moertel et al.^44^ synchronous prior cancers were defined as those that occurred within 6 months before the index NSCLC diagnosis. First, a Cox proportional hazards model was constructed to calculate the hazard ratio (HR) and 95% confidence interval (CI) of all-cause mortality in the Prior Cancer Group relative to the No Prior Cancer Group; this model examined all prior cancers within the OCR records linking with claims data, regardless of their diagnostic time intervals before the index NSCLC diagnosis. The model adjusted for the following covariates at the index NSCLC diagnosis: age, sex, method of cancer detection, treatment, body mass index, Barthel Index, CCI, interstitial lung disease, tyrosine kinase inhibitor use, smoking status, diagnosis year, and Area Deprivation Index. Next, we constructed additional Cox proportional hazards models to examine the impact of prior cancer on survival when the diagnostic time intervals were limited to within 1, 3, 5, 10, and 15 years before the index NSCLC diagnosis. These analyses were also stratified according to the index NSCLC stage. In each NSCLC stage, patients whose prior cancer occurred within the stipulated diagnostic time interval were categorized into the Prior Cancer Group, and patients without any prior cancer or whose prior cancer did not occur within the stipulated diagnostic time interval were categorized into the No Prior Cancer Group.

To examine the impact of prior cancers according to their characteristics, we constructed 3 Cox proportional hazards models. In Model 1, prior cancers were divided into smoking-related and non–smoking-related cancers. In Model 2, prior cancers were divided into those with better prognosis and poorer prognosis. In Model 3, prior cancers were categorized according to their stage at diagnosis.

Statistical significance was set at 5% (two-sided). All analyses were performed using STATA version 16 (Stata Corporation, College Station, TX, USA).

### Sensitivity analysis

Previous studies have reported that lung cancer patients with prior cancer do not have significantly poorer survival than those without prior cancer^12–14^. A possible explanation for those observations is that cancer patients with prior cancer may have longer lead times before a new cancer diagnosis (i.e., earlier detection) due to more frequent screening or prompt healthcare-seeking behavior for potential tumor symptoms. A previous study reported that the estimated mean lead times (MLTs) are 3.4, 1.1, and 1.1 months for lung cancer patients in stages I/II, III, and IV, respectively, under the assumption that only patients with prior cancer are susceptible to lead time bias^45^. To investigate the robustness of our results, we considered lead time bias in the Prior Cancer Group using 1 × MLT, 2 × MLT, and 3 × MLT. The survival analysis was then performed by subtracting the various MLT durations from survival time in the Prior Cancer Group according to index NSCLC stage^45,46^. In this sensitivity analysis, we regarded localized cancers as stage I/II, regional cancers as stage III, and distant cancers as stage IV.

### Ethics approval and consent to participate

This study was approved by the Kyoto University Graduate School of Medicine Ethics Committee (Approval No. R1808) and the Research Ethics Committee of Osaka International Cancer Institute (Approval No. 19143). The dataset was provided by the OCR with no personally identifiable information, and was processed independently in compliance with the Act on Promotion of Cancer Registries of Japan. Both ethics committees waived the need for informed consent in accordance with the Japanese government’s Ethical Guidelines for Medical and Health Research Involving Human Subjects, which allow for the opt-out approach for the secondary use of existing data. The study was performed in accordance with the ethical standards established in the Declaration of Helsinki.

## Results

### Baseline characteristics of the patients

**Figure 1:**
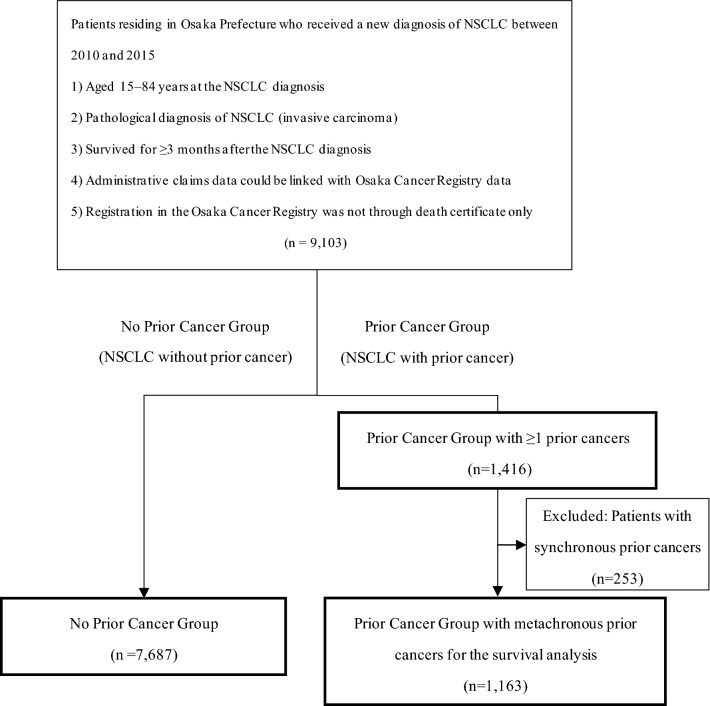
Flow diagram of patient selection. Abbreviation: NSCLC, non-small cell lung cancer.

The patient selection process is presented in Fig. 1. We first identified 9,103 index NSCLC patients who met the inclusion criteria. Among these, 1416 (15.6%) patients had 1 prior cancer or more. For the survival analysis, we excluded 253 patients with synchronous prior cancers. Table 1 summarizes the baseline characteristics of the NSCLC patients. In the No Prior Cancer Group, the median age (interquartile range) was 70 (64–75) years and women comprised 34.7% of the patients. In the Prior Cancer Group, the median age (interquartile range) was 73 (67–77) years and women comprised 29.5% of the patients. The median survival time (interquartile range) was 24.0 (12.9–46.2) months in the No Prior Cancer Group and 29.0 (15.9–47.0) months in the Prior Cancer Group.

**Table 1:** Baseline characteristics of the patients according to prior cancer status.

NSCLC patients n = 9103	No Prior Cancer Group^a^ n = 7687		Prior Cancer Group^b^ n = 1416	
	n	%	n	%
Sex				
Male	5020	65.3	998	70.5
Female	2667	34.7	418	29.5
Median age (IQR), years	70 (64–75)		73 (67–77)	
Age groups				
15–24 years	0	0.0	0	0.0
25–34 years	20	0.3	0	0.0
35–44 years	131	1.7	5	0.4
45–54 years	448	6.0	28	2.0
55–64 years	1520	20.0	190	13.4
65–74 years	3434	45.0	617	43.6
75–84 years	2134	27.8	576	40.7
Histology				
Adenocarcinoma	5005	65.1	884	62.4
Squamous cell carcinoma	2019	26.3	415	29.3
Adenosquamous carcinoma	79	1.0	32	2.3
Large cell carcinoma	275	3.6	55	3.9
NSCLC-not otherwise specified	309	4.0	30	2.1
NSCLC stage				
Localized	2661	34.6	800	56.5
Regional	1944	25.3	310	21.9
Distant	3008	39.1	288	20.3
Other/unknown	74	1.0	18	1.3
Method of cancer detection				
Screening and medical check-up	1087	14.1	77	5.4
Incidental detection^c^	2264	29.5	982	69.4
Other/unknown	4336	56.4	357	25.2
Treatment				
Radiotherapy only	361	4.7	80	5.7
Chemotherapy only	2032	26.4	206	14.6
Chemoradiotherapy	1086	14.1	107	7.6
Surgery only	2356	30.7	717	50.6
Surgery + chemotherapy and/or radiotherapy	1356	17.6	183	12.9
Other/unknown	496	6.5	123	8.7
Tyrosine kinase inhibitor use				
No	6531	85.0	1330	93.9
Yes	1156	15.0	86	6.1
Barthel index				
Mild or no dependence	7211	93.8	1357	95.8
Moderate dependence	167	2.2	14	1.0
Severe dependence	300	3.9	42	3.0
Unknown	9	0.1	3	0.2
Body mass index, kg/m^2^				
< 18.5	967	12.6	220	15.5
18.5–24.9	5027	65.4	917	64.8
25–29.9	1360	17.7	243	17.2
≥ 30	174	2.3	25	1.8
Unknown	159	2.1	11	0.8
Charlson comorbidity index score				
0	5656	73.6	1008	71.2
1	1276	16.6	220	15.5
≥ 2	755	9.8	188	13.3
Interstitial lung disease				
No	7426	96.6	1372	96.9
Yes	261	3.4	44	3.1
Smoking status				
No	2419	31.5	451	31.9
Yes	4918	64.0	906	64.0
Unknown	350	4.6	59	4.2
Area deprivation index				
Q1	2443	31.8	485	34.3
Q2	1592	20.7	302	21.3
Q3	1833	23.9	321	22.7
Q4	1772	23.1	298	21.1
Unknown	47	0.6	10	0.7
Vital status				
Alive	3672	47.8	743	52.5
Dead	4015	52.2	673	47.5
MST (IQR), months	24.0 (12.9–46.2)		29.0 (15.9–47.0)	

*IQR* Interquartile range, *MST* Median survival time, *NSCLC* Non-small cell lung cancer.

^a^Patients without any history of prior cancer.

^b^Patients with a history of prior cancer.

^c^Incidental detection during follow-up examination for another disease.

### Characteristics of prior cancer

The characteristics of prior cancer in the Prior Cancer Group are shown in Supplementary Table 3. The majority of patients in the Prior Cancer Group had only 1 prior cancer before the index NSCLC in both sexes. The cumulative proportion of patients whose most recent prior cancer was diagnosed within 5 years before the index NSCLC diagnosis was 62.1% (64.1% in male patients and 56.6% in female patients). The most common and least common prior cancer stages were localized and distant, respectively. The most common prior cancer sites were stomach (26.7%), prostate (14.9%), and colon (13.7%) among male patients; and breast (31.3%), stomach (15.8%), and colon (9.6%) among female patients. Smoking-related cancers accounted for 75.1% of all prior cancers in male patients and 48.8% of all prior cancers in female patients.

### Impact of prior cancer on survival according to diagnostic time interval and index NSCLC stage

**Figure 2:**
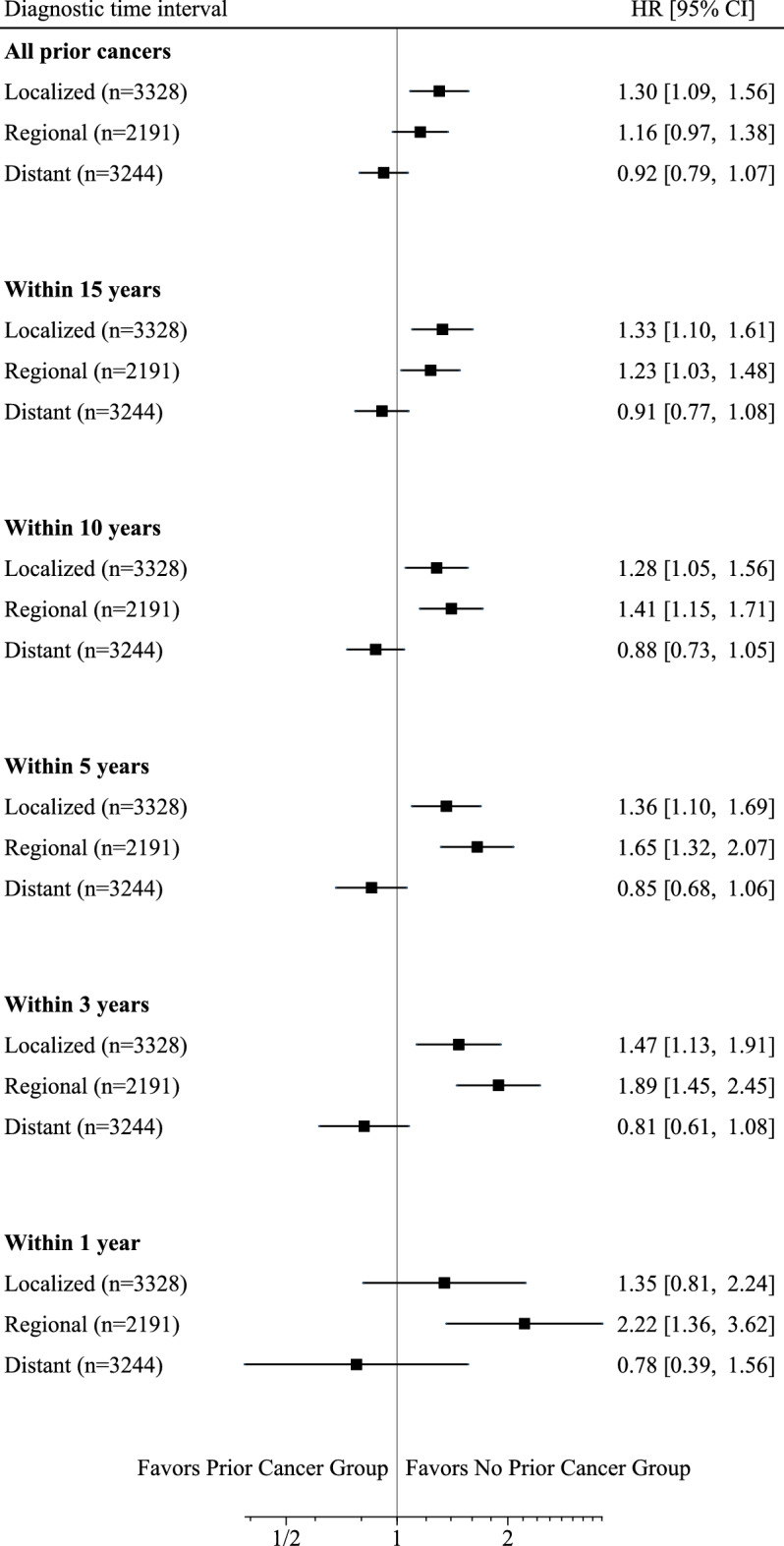
Impact of prior cancer on survival in NSCLC patients according to diagnostic time interval and index NSCLC stage. HRs were calculated using Cox proportional hazards models on all-cause mortality in NSCLC patients stratified by diagnostic time interval (between the most recent prior cancer and the index cancer) and index NSCLC stage. The No Prior Cancer Group was used as the reference category. For all diagnostic time intervals in these models, there were 3328 localized NSCLC patients, 2191 regional NSCLC patients, and 3244 distant NSCLC patients. Abbreviations: CI, confidence interval; HR, hazard ratio; NSCLC, non-small cell lung cancer.

The results of the Cox proportional hazards analyses according to diagnostic time interval are presented in Table 2. The adjusted HR of the Prior Cancer Group (ref: No Prior Cancer Group) for all-cause mortality was 1.07 (95% CI: 0.97–1.17), regardless of diagnostic time interval between the most recent prior cancer and the index NSCLC. When the diagnostic time intervals were limited to 3 and 5 years, the mortality HRs of the Prior Cancer Group were 1.23 (95% CI: 1.06–1.43) and 1.18 (1.04–1.33), respectively. When the diagnostic time intervals were limited to 1, 10, and 15 years, the Prior Cancer Group did not show any significantly higher hazards for mortality than the No Prior Cancer Group (Table 2). Area Deprivation Index scores were not associated with prognosis. When including all prior cancers regardless of diagnostic time interval in the analysis, the Prior Cancer Group was not significantly associated with mortality in regional and distant NSCLC patients, but had a significantly higher mortality hazard in localized NSCLC patients (Fig. 2). Among the limited diagnostic time intervals, the Prior Cancer Group had consistently higher mortality hazards in localized and regional NSCLC patients (except for diagnostic time intervals of 1 year), but not in distant NSCLC patients.

**Table 2:** Impact of prior cancer on survival in NSCLC patients according to diagnostic time interval.

Diagnostic time interval												
n = 8850	Within 1 year (n = 89)	*P*	Within 3 years (n = 338)	*P*	Within 5 years (n = 626)	*P*	Within 10 years (n = 904)	*P*	Within 15 years (n = 1010)	*P*	Any prior cancer^a^ (n = 1163)	*P*
	HR (95% CI)		HR (95% CI)		HR (95% CI)		HR (95% CI)		HR (95% CI)		HR (95% CI)	
No Prior Cancer Group^b^	Ref.		Ref.		Ref.		Ref.		Ref.		Ref.	
Prior Cancer Group^c^	1.32 (0.97–1.80)	0.077	1.23 (1.06–1.43)	0.007	1.18 (1.04–1.33)	0.009	1.10 (0.99–1.22)	0.092	1.09 (0.99–1.21)	0.082	1.07 (0.97–1.17)	0.176

HRs were calculated using Cox proportional hazards models on all-cause mortality in NSCLC patients stratified by diagnostic time interval (1, 3, 5, 10, and 15 years) between the most recent prior cancer and the index cancer.

The models adjusted for the following covariates measured at the index NSCLC diagnosis: age divided by 10, sex, method of cancer detection, treatment, body mass index, Barthel Index, Charlson Comorbidity Index, interstitial lung disease, tyrosine kinase inhibitor use, smoking status, diagnosis year, and Area Deprivation Index. The No Prior Cancer Group was used as the reference category.

*CI* Confidence interval, *HR* Hazard ratio, *NSCLC* non-small cell lung cancer.

^a^Prior cancer regardless of diagnostic time interval.

^b^Patients without any history of prior cancer prior cancer.

^c^Patients with a history of prior cancer.

### Impact of prior cancer on survival according to prior cancer characteristics

**Figure 3:**
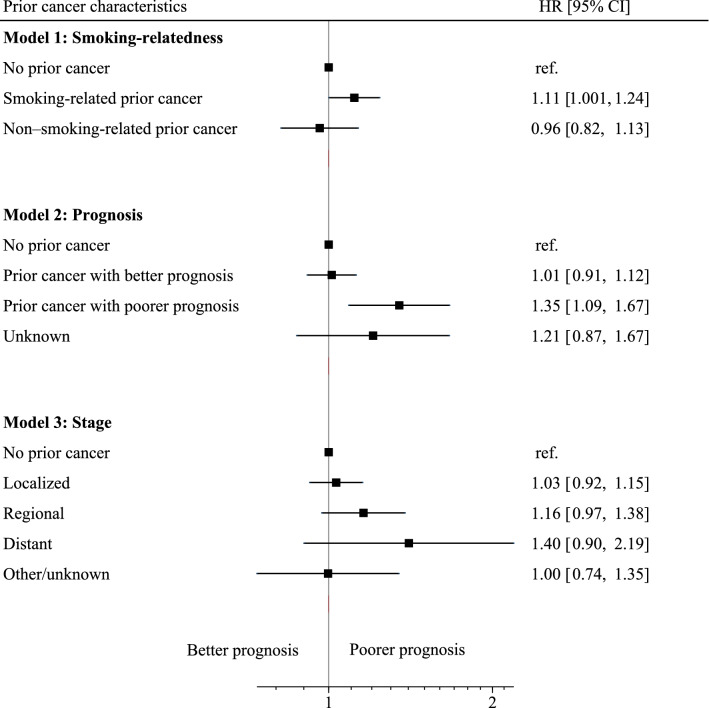
Impact of prior cancer on survival in NSCLC patients according to prior cancer characteristics. HRs were calculated using 3 Cox proportional hazards models on all-cause mortality in NSCLC patients. In Model 1, NSCLC patients were divided into no prior cancer, smoking-related prior cancer, and non–smoking-related prior cancer. In Model 2, NSCLC patients were divided into no prior cancer, prior cancer with better prognosis, and prior cancer with poorer prognosis. In Model 3, NSCLC patients were divided into no prior cancer, localized prior cancer, regional prior cancer, and distant prior cancer. The No Prior Cancer Group was used as the reference category. Abbreviations: CI, confidence interval; HR, hazard ratio; NSCLC, non-small cell lung cancer.

Additional analyses were performed to examine the heterogeneous effects of prior cancers according to their characteristics (Fig. 3). Here, we categorized prior cancers according to smoking-relatedness (Model 1), prognosis (Model 2), and stage at diagnosis (Model 3). Smoking-related prior cancers (HR: 1.11; 95% CI: 1.001–1.22) in Model 1 and prior cancers with poorer prognosis (HR: 1.35; 95% CI: 1.09–1.67) in Model 2 showed higher mortality hazards than the No Prior Cancer Group. In Model 3, the mortality hazard appeared to increase together with prior cancer stage, but this relationship was not significant.

### Sensitivity analysis

To assess the robustness of our results, we considered lead time bias in the Prior Cancer Group. The Prior Cancer Group was not significantly associated with mortality in distant NSCLC patients, but had a significantly higher mortality hazard in localized NSCLC patients (Table 3). These findings were consistent with the results of the main analysis.

**Table 3:** Sensitivity analysis of the impact of prior cancer on survival in NSCLC patients for different MLT durations.

MLT duration	Localized			Regional			Distant		
	HR (95% CI)	*P*	MST (IQR), months	HR (95% CI)	*P*	MST (IQR), months	HR (95% CI)	*P*	MST (IQR), months
1 × MLT	1.40 (1.16–1.68)	< 0.001	43.0 (23.6–51.9)	1.20 (1.00–1.43)	0.047	24.6 (14.5–46.0)	0.87 (0.74–1.03)	0.105	13.7 (7.2–24.3)
2 × MLT	1.47 (1.21–1.78)	< 0.001	42.2 (23.1–51.0)	1.20 (0.99–1.43)	0.051	24.7 (14.8–46.0)	0.87 (0.74–1.03)	0.103	13.6 (7.2–24.3)
3 × MLT	1.49 (1.21–1.59)	0.003	41.2 (22.7–51.0)	1.21 (1.00–1.45)	0.047	24.5 (14.6–46.0)	0.89 (0.75–1.06)	0.189	13.6 (7.2–24.3)

The estimated MLT durations were set at 3.4, 1.1, and 1.1 months for patients with localized (stage I/II), regional (stage III), and distant (stage IV) NSCLC, respectively. HRs were calculated using Cox proportional hazards models on all-cause mortality in NSCLC patients after subtracting the various MLT durations from survival time in the Prior Cancer Group.

*CI* Confidence interval, *HR* Hazard ratio, *IQR* Interquartile range, *MLT* Mean lead time, *MST* Median survival time, *NSCLC* Non-small cell lung cancer.

## Discussion

Using a large dataset consisting of cancer registry data linked with claims data, this study analyzed the impact of prior cancer on survival in newly diagnosed NSCLC patients. Our analysis provides new insight into this relationship with consideration to patient characteristics, cancer characteristics, and diagnostic time intervals. Our results were consistent when incorporating the potential for the lead-time bias among the cancer survivors.

We found that longer diagnostic time intervals between the prior cancers and index NSCLC were generally characterized by smaller effect sizes (mortality HRs). For diagnostic time intervals of 3 and 5 years, prior cancer was significantly associated with a higher mortality hazard. However, this relationship was not observed for other diagnostic time intervals. Although many clinical trials employ a 5-year exclusion window for prior cancers without a clear rationale^7,47^, our findings indicate that this criterion may be justified. Nevertheless, the 5-year exclusion window may not be necessary for clinical trials involving patients with more advanced NSCLC as our analysis showed that prior cancer was not associated with prognosis in these patients. A possible explanation could be that regional/distant NSCLC is already associated with poorer prognosis, which may have a greater impact on survival than a history of prior cancer. Prior cancer may have little or no prognostic impact in patients with more advanced NSCLC. There is the growing prevalence of lung cancer patients with prior cancer and the cumulative proportions of the most recent prior cancers diagnosed within 5 years before the lung cancer diagnosis were 69.4% (male) and 65.0% (female)^4^. These may indicate that relatively large numbers of lung cancer patients will not be eligible for clinical trials by the 5-year exclusion window.

Our findings from the main analysis were supported by the sensitivity analysis that accounted for potential lead time bias^45^, which was often neglected in previous studies on the association between prior cancer and prognosis in NSCLC patients^12–14^. Our study therefore provides robust evidence that the prognostic impact of prior cancers is influenced by the index NSCLC stage.

In addition to assessing the prognostic implications of prior cancer according to diagnostic time interval, we also examined the heterogeneous effects of prior cancer according to their characteristics. Here, we found that smoking-related prior cancers had a significantly negative impact on survival in newly diagnosed NSCLC patients. This may be because smoking-related cancer survivors have a higher risk of being cigarette smokers, which is an important prognostic factor for NSCLC^17,48^. In addition, prior cancers with poorer prognosis were associated with higher mortality for the index NSCLC. This suggests that the sites of prior cancer and their associated survival rates should be taken into account when evaluating their prognostic impact.

### Strengths and limitations

A strength of this study was the inclusion of diagnostic time intervals between the prior cancers and index lung cancer, and we propose that these intervals should be considered in the eligibility criteria for clinical trials on NSCLC patients. Another strength was the categorization of prior cancer according to its characteristics, which enabled a more in-depth analysis of which aspects of prior cancer could affect survival. Furthermore, by linking cancer registry data with claims data, we were able to account for many known prognostic factors (e.g., performance in activities of daily living, comorbidities, and smoking status) that are frequently absent from registry-based studies.

Our study has several limitations. First, we did not have access to information on TNM classification, gene mutations, and ECOG-PS, which are often used in clinical practice. As alternatives to these indicators, we used SEER summary staging, tyrosine kinase inhibitor use, and the Barthel Index due to their availability in the claims data. Second, although the OCR has been in operation since 1962 and has accumulated cancer incidence data from over a million patients in Osaka Prefecture, our findings should be validated using data from other regions or countries. Third, our study was conducted using cancer registry data linked with administrative claims data from approximately half of all cancer patients within Osaka Prefecture. Therefore, the study population may not be representative of the entire population in the study region, and could be susceptible to selection bias.

Cancer treatment should be provided to patients with prior cancer based on an empirical understanding of their possible prognostic relevance. As the number of cancer survivors increases steadily, there is a need to increase their representation in clinical cancer research in order to generate evidence for their treatment and improve the generalizability of results.

In conclusion, NSCLC patients with prior cancer do not have an invariably higher risk of mortality than those without prior cancer. NSCLC patients with prior cancer should be considered for inclusion in clinical trials, especially for studies on regional and distant NSCLC. More inclusive clinical trials are required to better inform treatment strategies, and our findings underscore the need to revisit the eligibility criteria for cancer survivors in clinical research.

## Supplementary Information

Supplementary Information.

## Data Availability

The datasets analyzed during the current study are available from the corresponding author on reasonable request.

## Abbreviations

HR: Hazard ratio
CI: Confidence interval
CCI: Charlson comorbidity index
ECOG-PS: Eastern Cooperative Oncology Group-Performance Status
ICD-10: International classification of diseases, tenth revision
NSCLC: Non-small cell lung cancer
OCR: Osaka Cancer Registry
SEER: Surveillance, epidemiology, and end results
MLT: Mean lead times
