# Supplementary material for: Survival in non-small cell lung cancer patients with versus without prior cancer
Source: Sci Rep. 2023 Mar 15;13:4269. doi: 10.1038/s41598-023-30850-2 (PMC10017802; doi:10.1038/s41598-023-30850-2)
Supplement: Supplementary file 1 — Supplementary Information. [file 41598_2023_30850_MOESM1_ESM.docx]

# SUPPLEMENTARY MATERIALS

**TITLE:**

**Survival in Non-Small Cell Lung Cancer Patients With Versus Without Prior Cancer**

AUTHORS

Akira Sato, MD, MPH, Toshitaka Morishima, MD, PhD, Masato Takeuchi, MD, MPH, PhD, Kayo Nakata, MD, PhD, Koji Kawakami, MD, PhD, and Isao Miyashiro, MD, PhD

**Corresponding author**: Toshitaka Morishima, MD, PhD

Cancer Control Center, Osaka International Cancer Institute, 3-1-69 Otemae, Chuo-ku, Osaka, Japan 541-8567, E-mail: morishima.t@oici.jp

**List of supplementary materials**

**Supplementary Table 1. Definition of prior cancer sites according to ICD-10 codes**

**Supplementary Table 2. Classification of prior cancers according to relative survival rates**

**Supplementary Table 3. Prior cancer characteristics in the Prior Cancer Group**

| **Supplementary Table 1. Definition of prior cancer sites according to ICD-10 codes** |
| --- |
| All sites (C00–C96.x), Mouth and pharynx (C00–C14.x), Esophagus (C15.x), Stomach (C16.x), Small intestine (C17.x), Colon (C18.x), Rectum (C19.x–C20.x), Liver (C22.x), Gallbladder and bile duct (C23.x–C24.x), Pancreas (C25.x), Larynx (C32.x), Trachea (C33.x), Bronchus and lung (C34.x), Thymus (C37.x), Heart, mediastinum, and pleura (C38.x), Bones, joints, and articular cartilage (C40–C41.x), Melanoma of skin (C43.x), Other skin (C44.x), Mesothelioma (C45.x), Breast (C50.x), Uterus (C53.x–C55.x), Cervix uteri (C53.x), Corpus uteri (C54.x), Ovary (C56.x), Prostate (C61.x), Testis (C62.x), Other and unspecified male genital organs (C63.x), Kidney, except renal pelvis (C64.x), Renal pelvis (c65.x), Ureter (C66.x), Bladder (C67.x), Brain and central nervous system (C70.x–C72.x), Meninges (C70.x), Brain (C71.x), Spinal cord, cranial nerves, and other parts of the central nervous system (C72.x), Thyroid gland (C73.x), Adrenal gland (C74.x), Hodgkin lymphoma (C81.x), Non-Hodgkin lymphoma (C82.x–C86.x, C96.x), Immunoproliferative diseases (C88.x), Myeloma (C90.x), Lymphoid leukemia (C91.x), Acute myeloid leukemia (C92.0), Myeloid and monocytic leukemia (C92.x–C93.x), Other leukemia unspecified (C94.x–C95.x), and other (C21.x, C26.x, C30.x, C31.x, C39.x, C46.x–C49.x, C51.x, C52.x, C57.x, C58.x, C60, C68, C69, C75–C80.x; unknown primary sites).^31^ |

Abbreviation: ICD-10, International Classification of Diseases, Tenth Revision.

| **Supplementary Table 2. Classification of prior cancers according to relative survival rates** | | |
| --- | --- | --- |
| Classification | Male  Prior cancer site (ICD-10 codes) | Female  Prior cancer site (ICD-10 codes) |
| Poorer prognosis (<50%) | Pancreas (C25), Liver (C22), Multiple myeloma (C88–C90), Lung (C33–C34), Gallbladder (C23–C24), Leukemia (C91–C95), Brain and central nervous system (C70–C72, C75.1–C75.3), Esophagus (C15), Lip, oral cavity, and pharynx (C00–C14), Malignant lymphoma (C81–C85, C96) | Pancreas (C25), Liver (C22), Multiple myeloma (C88–C90), Gallbladder (C23–C25), Leukemia (C91–C95), Brain and central nervous system (C70–C72, C75.1–C75.3), Lung (C33–C34), Esophagus (C15), Ovary (C56) |
| Better prognosis (≥50%) | Kidney, renal pelvis, and ureter (C64–C66, C68), Rectum (C19–C20), Stomach (C16), Colon (C18), Larynx (C32), Bladder (C67), Prostate (C61), Skin (C43–C44), Thyroid (C73) | Malignant lymphoma (C81–C85, C96), Lip, oral cavity, and pharynx (C00–C14), Kidney, renal pelvis, and ureter (C64–C66, C68), Stomach (C16), Colon (C18), Bladder (C67), Rectum (C19–C20), Cervix uteri (C53), Larynx (C32), Corpus uteri (C54), Breast (C50), Skin (C43–C44), Thyroid (C73) |
| Prior cancers were categorized into “better prognosis” or “poorer prognosis” using reported median 10-year relative survival rates.^43^ Cancers not included above were categorized as having “unknown” survival.  Abbreviation: ICD-10, International Classification of Diseases, Tenth Revision. | | |

| **Supplementary Table 3. Prior cancer characteristics in the Prior Cancer Group** | | | | | | |
| --- | --- | --- | --- | --- | --- | --- |
| Prior Cancer Group  n=1,416 | Male  n=998 | | Female  n=418 | | Total | |
|  | n | % | n | % | n | % |
| Number of prior cancers before the index cancer |  |  |  |  |  |  |
| 1 | 857 | 85.9 | 368 | 88.0 | 1,225 | 86.5 |
| 2 | 128 | 12.8 | 47 | 11.2 | 175 | 12.4 |
| 3 | 13 | 1.3 | 3 | 0.7 | 16 | 1.1 |
| Diagnostic time interval^a^ |  |  |  |  |  |  |
| <0.5 years | 178 | 17.8 | 75 | 17.9 | 253 | 17.9 |
| 0.5–0.9 years | 64 | 6.4 | 25 | 6.0 | 89 | 6.3 |
| 1–2.9 years | 229 | 22.9 | 70 | 16.7 | 299 | 21.1 |
| 3–4.9 years | 171 | 17.1 | 67 | 16.0 | 238 | 16.8 |
| 5–9.9 years | 193 | 19.3 | 85 | 20.3 | 278 | 19.6 |
| 10–14.9 years | 67 | 6.7 | 39 | 9.3 | 106 | 7.5 |
| ≥15 years | 96 | 9.6 | 57 | 13.6 | 153 | 10.8 |
| Prior cancer stage^b^ |  |  |  |  |  |  |
| Localized | 664 | 66.5 | 256 | 61.2 | 920 | 65.0 |
| Regional | 220 | 22.0 | 116 | 27.8 | 336 | 23.7 |
| Distant | 46 | 4.6 | 14 | 3.3 | 60 | 4.2 |
| Other/Unknown | 68 | 6.8 | 32 | 7.7 | 100 | 7.1 |
| Prior cancer site^b^ |  |  |  |  |  |  |
| Mouth and pharynx | 50 | 5.0 | 12 | 2.9 | 62 | 4.4 |
| Esophagus | 53 | 5.3 | 4 | 1.0 | 57 | 4.0 |
| Stomach | 266 | 26.7 | 66 | 15.8 | 332 | 23.4 |
| Small intestine | 10 | 1.0 | 2 | 0.5 | 12 | 0.8 |
| Colon | 137 | 13.7 | 40 | 9.6 | 177 | 12.5 |
| Rectum | 68 | 6.8 | 26 | 6.2 | 94 | 6.6 |
| Liver | 38 | 3.8 | 10 | 2.4 | 48 | 3.4 |
| Gallbladder and bile duct | 3 | 0.3 | 1 | 0.2 | 4 | 0.3 |
| Pancreas | 15 | 1.5 | 2 | 0.5 | 17 | 1.2 |
| Larynx | 41 | 4.1 | 4 | 1.0 | 45 | 3.2 |
| Thymus | 1 | 0.1 | 1 | 0.2 | 2 | 0.1 |
| Bones, joints, and articular cartilage | 1 | 0.1 | 0 | 0.0 | 1 | 0.1 |
| Melanoma of skin | 0 | 0.0 | 1 | 0.2 | 1 | 0.1 |
| Other skin | 16 | 1.6 | 3 | 0.7 | 19 | 1.3 |
| Mesothelioma | 1 | 0.1 | 0 | 0.0 | 1 | 0.1 |
| Breast | 1 | 0.1 | 131 | 31.3 | 132 | 9.3 |
| Cervix uteri | 0 | 0.0 | 23 | 5.5 | 23 | 1.6 |
| Corpus uteri | 0 | 0.0 | 13 | 3.1 | 13 | 0.9 |
| Uterus, part unspecified | 0 | 0.0 | 1 | 0.2 | 1 | 0.1 |
| Ovary | 0 | 0.0 | 9 | 2.2 | 9 | 0.6 |
| Prostate | 149 | 14.9 | 0 | 0.0 | 149 | 10.5 |
| Testis | 6 | 0.6 | 0 | 0.0 | 6 | 0.4 |
| Other and unspecified male genital organs | 1 | 0.1 | 0 | 0.0 | 1 | 0.1 |
| Kidney, except renal pelvis | 24 | 2.4 | 10 | 2.4 | 34 | 2.4 |
| Renal pelvis | 4 | 0.4 | 1 | 0.2 | 5 | 0.4 |
| Ureter | 9 | 0.9 | 1 | 0.2 | 10 | 0.7 |
| Bladder | 44 | 4.4 | 4 | 1.0 | 48 | 3.4 |
| Meninges | 1 | 0.1 | 0 | 0.0 | 1 | 0.1 |
| Brain | 1 | 0.1 | 0 | 0.0 | 1 | 0.1 |
| Spinal cord, cranial nerves, and other parts of central nervous system | 0 | 0.0 | 1 | 0.2 | 1 | 0.1 |
| Thyroid gland | 9 | 0.9 | 31 | 7.4 | 40 | 2.8 |
| Hodgkin lymphoma | 3 | 0.3 | 1 | 0.2 | 4 | 0.3 |
| Non-Hodgkin lymphoma | 26 | 2.6 | 11 | 2.6 | 37 | 2.6 |
| Myeloma | 3 | 0.3 | 0 | 0.0 | 3 | 0.2 |
| Lymphoid leukemia | 0 | 0.0 | 2 | 0.5 | 2 | 0.1 |
| Myeloid and monocytic leukemia | 1 | 0.1 | 1 | 0.2 | 2 | 0.1 |
| Other | 16 | 1.6 | 6 | 1.4 | 22 | 1.6 |
| Smoking-related prior cancer^c^ |  |  |  |  |  |  |
| Yes | 749 | 75.1 | 204 | 48.8 | 953 | 67.3 |
| No | 249 | 24.9 | 214 | 51.2 | 463 | 32.7 |
| Values are expressed as the number of patients and column percentage. ^a^ Interval between the most recent prior cancer and the index non-small cell lung cancer. ^b^ Most recently diagnosed prior cancer before the index non-small cell lung cancer. ^c^ Smoking-related prior cancers included cancers of the mouth, pharynx, larynx, lung, esophagus, stomach, liver, pancreas, kidney, urinary bladder, colorectum, uterine cervix, and acute myeloid leukemia. | | | | | | |
